# Plexiform’s perplexities: a tale of two plexiform neurofibromas

**DOI:** 10.1093/jscr/rjae486

**Published:** 2024-08-15

**Authors:** Aditya Sriharsha Pedaprolu, Rajesh Gattani, Suhas Jajoo, Venkatesh Rewale, Swati Deshpande, Priya Chatterjee, Mehak Fayyaz Semy

**Affiliations:** Department of General Surgery, Jawaharlal Nehru Medical College, Datta Meghe Institute of Higher Education and Research, Sawangi, Wardha, Maharashtra 442001, India; Department of General Surgery, Jawaharlal Nehru Medical College, Datta Meghe Institute of Higher Education and Research, Sawangi, Wardha, Maharashtra 442001, India; Department of General Surgery, Jawaharlal Nehru Medical College, Datta Meghe Institute of Higher Education and Research, Sawangi, Wardha, Maharashtra 442001, India; Department of General Surgery, Jawaharlal Nehru Medical College, Datta Meghe Institute of Higher Education and Research, Sawangi, Wardha, Maharashtra 442001, India; Department of General Surgery, Jawaharlal Nehru Medical College, Datta Meghe Institute of Higher Education and Research, Sawangi, Wardha, Maharashtra 442001, India; Department of Pathology, Jawaharlal Nehru Medical College, Datta Meghe Institute of Higher Education and Research, Sawangi, Wardha, Maharashtra 442001, India; Department of Medicine, Dr. DY Patil Hospital and Research Centre, Nerul, Navi Mumbai, Maharashtra 400706, India

**Keywords:** plexiform neurofibroma, von Recklinghausen’s disease, nerve tumor, neurofibromatosis, neurofibroma, café-au-lait macules

## Abstract

Plexiform neurofibroma (PF) is a rare benign variant belonging to a subtype of neurofibromatosis type 1 that forms bulging or deforming masses arising from the peripheral nerve sheath. These masses involve surrounding connective tissue or dermal layers, leading to multiple cutaneous changes and certain characteristic appearances. It is these appearances that aid in the diagnosis of PF. We have encountered two distinct patients diagnosed with this disorder. While one patient was clinically and pathologically confirmed for PF, the other had no characteristic cutaneous changes. The diagnosis was made with postoperative histopathology and confirmed with an immunohistochemical examination. There are various modalities in the management of PFs, with surgery being a mainstay in the treatment of disfiguring large PFs, especially in resource-restrained settings. In view of high recurrence rates, postoperative clinical follow-up is a must. This paper describes these patients’ typical and atypical clinical presentation and subsequent management.

## Introduction

Plexiform neurofibroma (PF), a rare subtype of neurofibromatosis type-1 (NF-1), involves multiple nerve bundles and leads to the formation of multiple neurocutaneous growths and alterations all over the body (neurofibromas). It comprises 5%–15% of patients with neurofibromatosis, causing developmental changes in the bone, skin, and nervous system. Mutation of the NF1 gene (codes for the protein neurofibromin), located on chromosome 17, is responsible for the formation of neurofibromas, café-au-lait macules, and freckles in the axillary or inguinal region (Crowe sign) [1, 2]. PFs can cause pain, neurological deficits, or even mood disorders resulting from disfigurement. The deforming masses involve surrounding connective tissues and skin folds, leading to a clinical description of a ‘bag of worms’ during palpation of lesions [3]. We intend to highlight two cases diagnosed with PF, exhibiting a notable disparity in their clinical manifestations. We analyze their clinical presentations, investigations leading to their diagnosis, and subsequent multidisciplinary management of this uncommon disorder.

## Case presentation

### Case one: typical presentation

**Figure 1:**
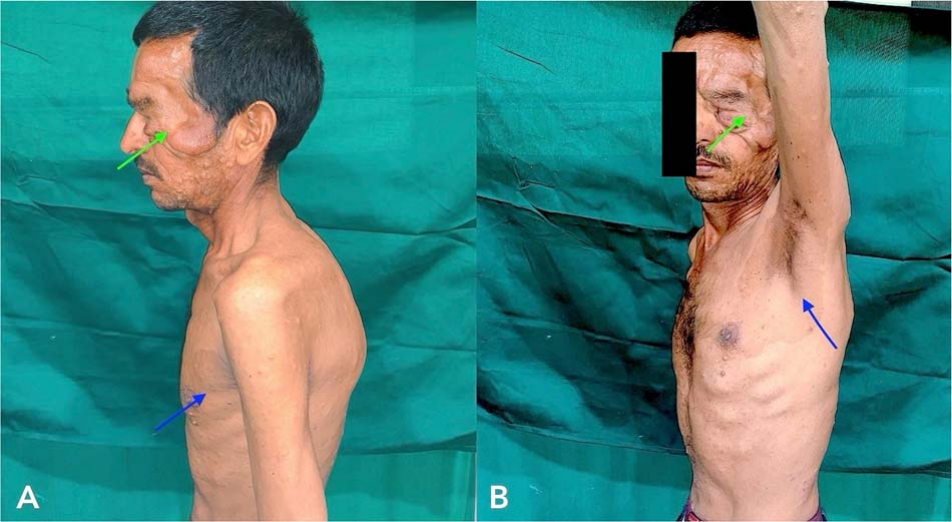
Clinical examination of the patient revealed localized Swelling over the left eyelid with consistency like a ‘Bag of worms’ and Café-au- lait macules and freckles over axillae (indicated with arrow).

A 43-year-old male patient came to our hospital with a gradually progressive, painless swelling involving his left eyelid, forehead, and cheek, along with multiple swellings all over his body for 37 years. He was diagnosed with PF 27 years ago and was operated on for facial swellings and fascia lata sling surgery for ptosis correction. However, the swellings have since recurred with ptosis. On clinical head-to-toe examination, café-au-lait macules and freckles over the axillae were identified (Fig. 1). Family history revealed that multiple relatives of his family have had similar presentations and complaints. He was advised to undergo genetic testing but denied it due to financial constraints.

**Figure 2:**
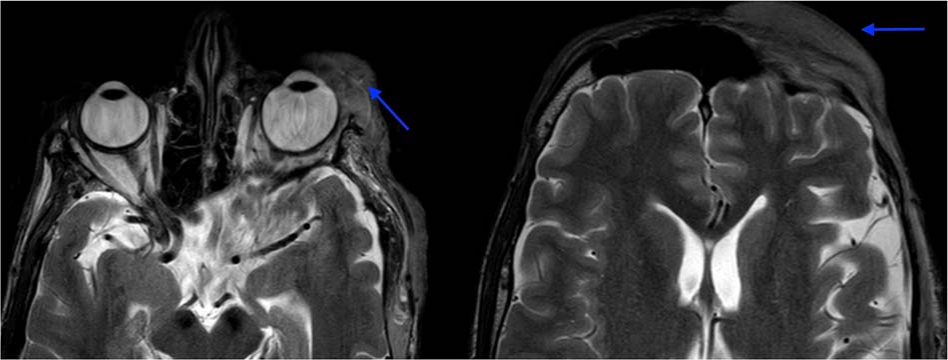
MRI orbit revealed enhancing skin thickening over the left frontal region, maxillary region, temporal region, supraorbital region, and over the left eyelids covering the eye.

**Figure 3:**
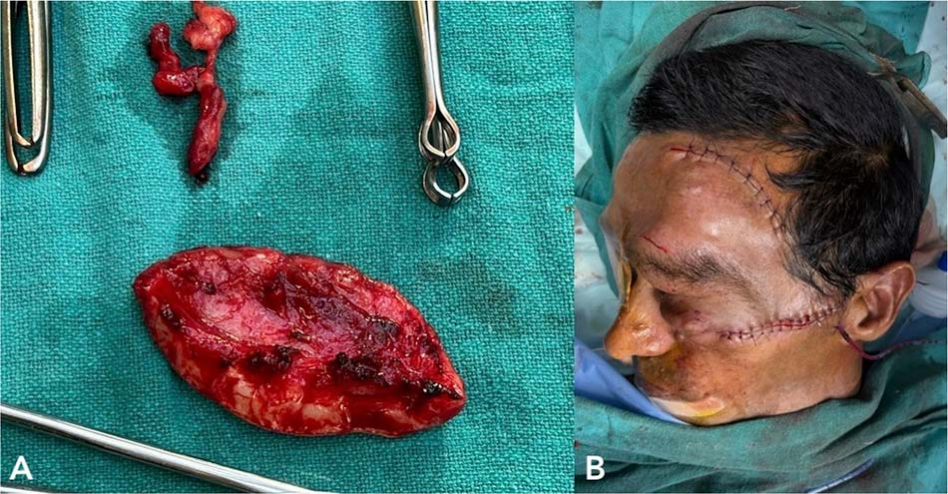
(A) shows excised neurofibromatosis tissue, which was sent for histopathology. (B) shows a postoperative image following closure.

MRI revealed multiple nodular skin lesions over the scalp, suggesting neurofibroma (Fig. 2). The patient was operated on for surgical debulking under general anesthesia. Intra-operatively, neurofibromatous tissue was meticulously identified and dissected, while preserving the underlying peri-orbital musculature (Fig. 3).

**Figure 4:**
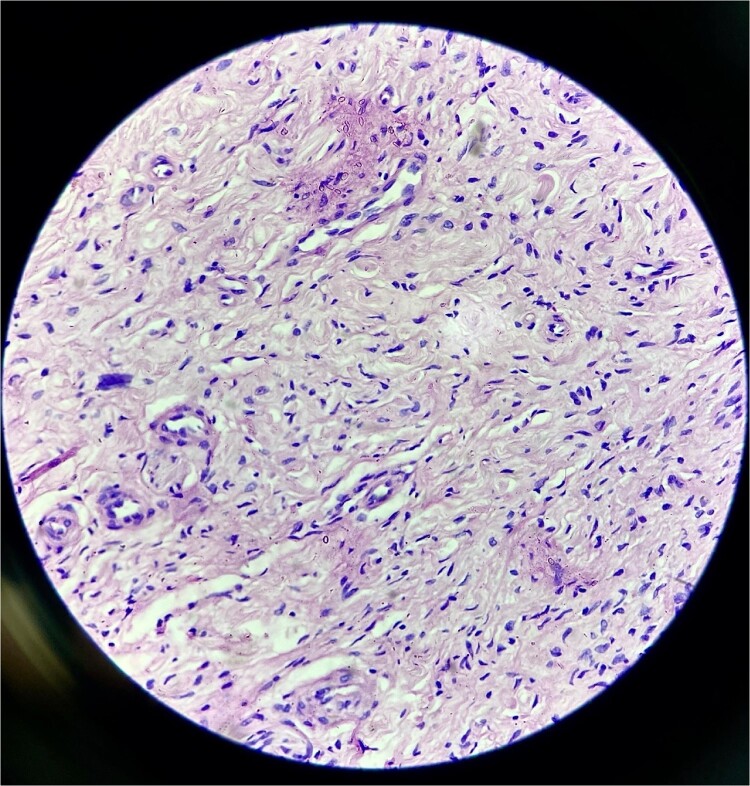
Histopathological examination revealed hypocellular proliferation of bland, slender to spindle-shaped cells with interspersed collagen.

Histopathology confirmed the diagnosis of PF (Fig. 4). The postoperative course in the hospital was uneventful. He has been kept on regular follow-ups every 6 months to monitor for recurrence or any neurological deficits. A follow-up with the ophthalmology department for correction of ptosis has also been planned.

### Case two: atypical presentation

**Figure 5:**
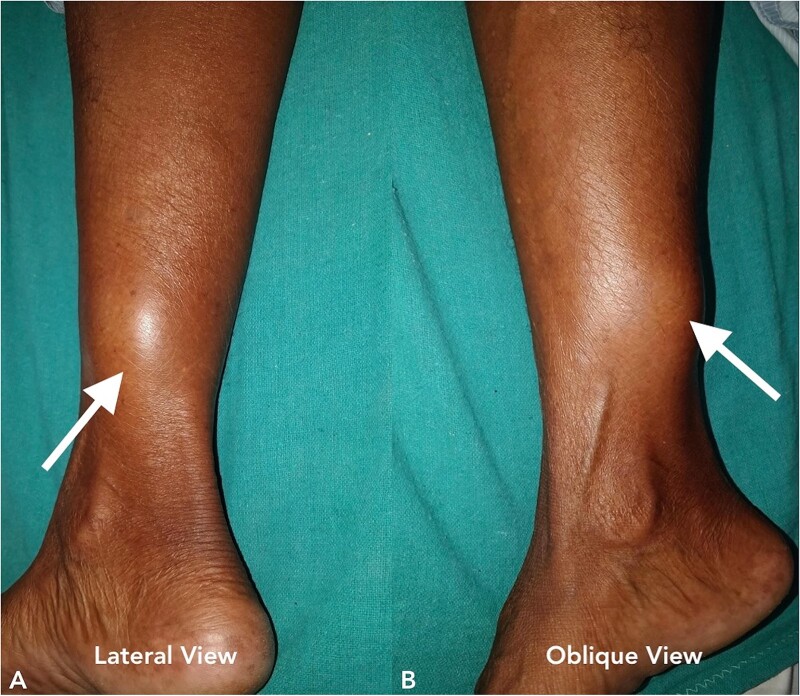
Clinical image of the patient’s swelling (marked with arrow) taken in lateral view (A) and oblique view (B).

A 70-year-old female presented to our hospital with chief complaints of a gradually progressive swelling over the inferior-medial aspect of a right lower limb for 20 years. This swelling was associated with an occasional sharp or burning type of pain. A local examination revealed a 5 × 5 cm smooth, firm, and non-mobile swelling (Fig. 5). The head-to-toe examination did not reveal any cutaneous or ophthalmic manifestations.

**Figure 6:**
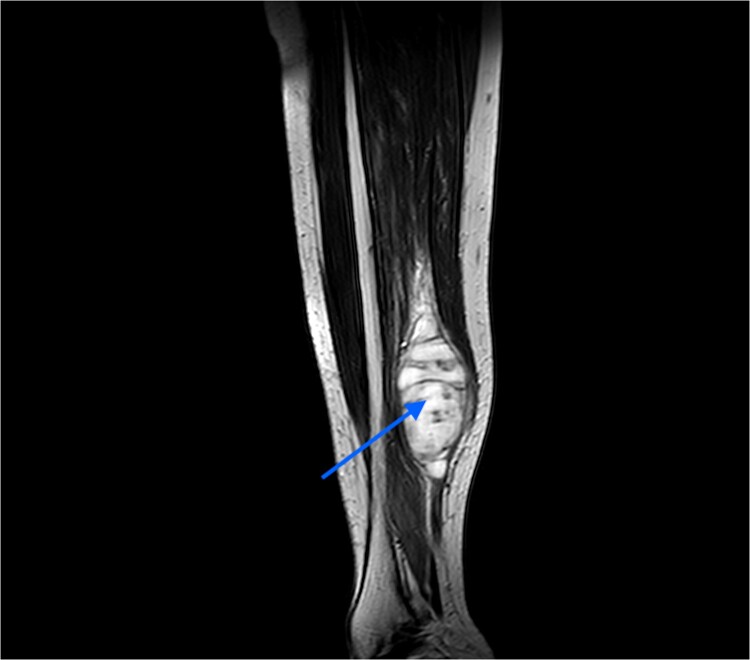
MRI image taken in coronal view, which was suggestive of a spindle-shaped mass lesion (indicated by arrow) located in the posterior compartment of the leg.

An MRI was done, which suggested a multi-lobulated spindle-shaped mass (Fig. 6).

**Figure 7:**
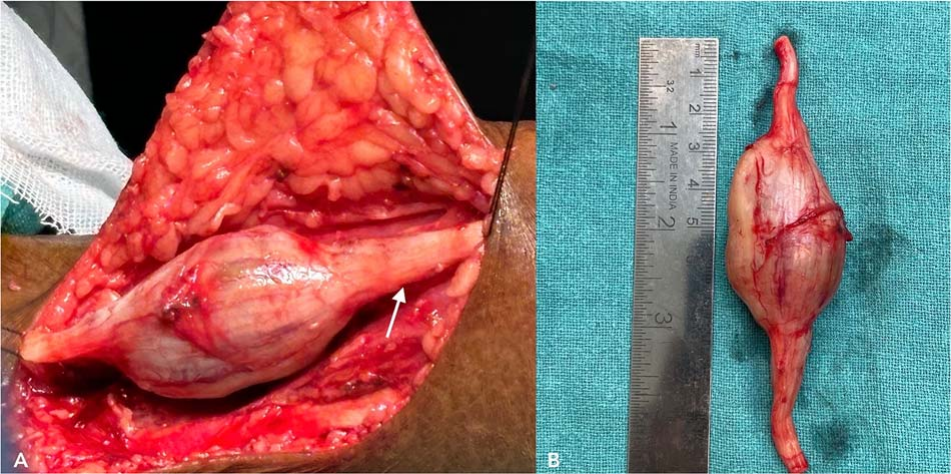
(A) shows an intra-operative image of the tumor, a part of the saphenous nerve (indicated by the arrow). (B) shows the excised specimen.

**Figure 8:**
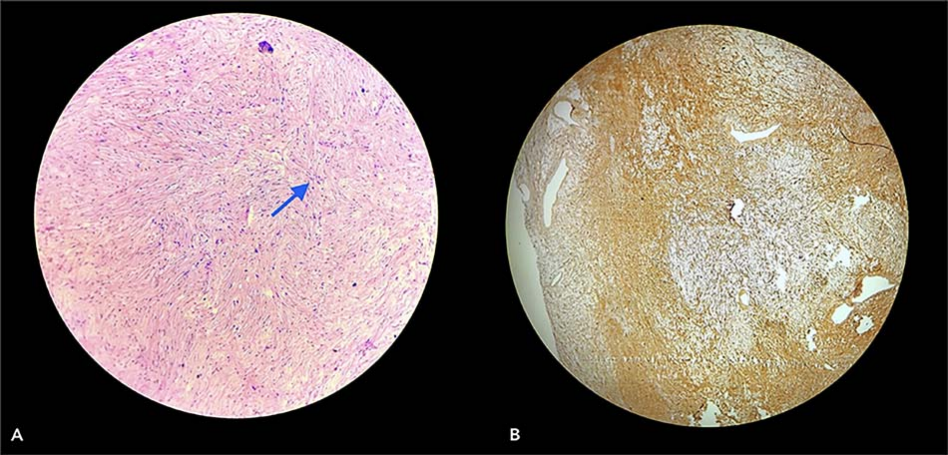
Section (A) shows wavy spindle cells with elongated nuclei and elongated cytoplasm within a fibrillary-type background with myxoid changes suggestive of PF. Section (B) of the IHC marker test shows scattered positivity in tumor cells, confirming the diagnosis. (S-100 = Positive and Ki67 = <10).

With due consent for a possible complication of postoperative neurological sensorimotor deficit, the patient was planned for surgical excision of the tumor under anesthesia. Intra-operatively, we found the tumor part of the saphenous nerve adherent to the surrounding muscle and underlying bone. A wide local excision was done with a 2 cm margin along the nerve from the tumor, and ends were ligated and cut with a scissor (Fig. 7). The excised specimen was sent for histopathology analysis, suggesting PF. This finding was perplexing, as the patient did not meet the clinical diagnostic criteria for PF. Immunohistochemical tests of S-100 and Ki67 were done, which confirmed the diagnosis. (Fig. 8).

Her postoperative period was uneventful, and she was eventually discharged and scheduled for bi-annual follow-up. Until recently, she didn’t develop any signs of palsy or neurological deficits. Her gait was normal, and she recovered without any complications.

## Discussion

Neurofibromatosis is an autosomal dominant genetic disorder with a 50% risk of transmission of the disease to their offspring. It has a variety of clinical presentations, from patients being either asymptomatic to others with large, invasive, and disfiguring lesions [4]. Other than the common cutaneous findings, such as café-au-lait spots, axillary freckles, lisch nodules in the iris (iris hamartomas), and skeletal dysplasia, can also be observed. Due to this variability, diagnosing this disorder can pose various challenges. During our course of treatment, the clinical presentation of our first patient did not suggest PN or even NF-1. Pathological co-relation and immunohistochemical biomarkers have helped confirm the diagnosis of PN. Besides S-100 protein or Ki67 markers, antibodies like calretinin, CD34, factor XIIIa, and CD56 can also diagnose and confirm neurofibroma [5, 6]. Investigations like MRI, karyotype analysis, and genetic and familial history of the patient with pedigree charting are needed for suspected cases of neurofibromatosis, which could aid in diagnosis.

Treatment for PF typically involves a multidisciplinary team, with surgery, radiation therapy, and close monitoring being the main approaches. Surgery has its challenges, including high vascularity of lesions and infiltration of these lesions into deeper planes. Recurrence post-surgery occurs in around 20% of cases, particularly in pediatric patients rather than adults. Another complication is the development of neurological deficits (such as foot drop, neuralgia, weakness over the operative region, and paraesthesia), which can be managed with surveillance, follow-up, and prophylactic physiotherapy [7]. Approximately 8%–12% of cases may progress to malignant peripheral nerve tumors, emphasizing the importance of vigilant follow-up and monitoring for signs, such as rapid growth and continuous pain or weakness [8]. Recent research has explored the administration of Interferon alpha or Selumetinib, with the latter receiving approval from the US Food and Drug Administration. Other drugs being tried include Interferon alpha, Cabozantinib, Mirdametinib, Trametinib, and Binimetinib, to name a few [9]. Results indicate significant functionality, especially in addressing inoperable tumors. However, further efforts are required to enable widespread implementation of these treatments [10, 11].

## Conclusion

We present two separate cases of patients diagnosed with PF. While the clinical presentations may vary, early diagnosis is crucial. Management depending on the operability of favorable outcomes is imperative. If surgical management is preferred, thorough preoperative planning, a multidisciplinary approach with the help of neurology, ophthalmology, or physiotherapy specialties, and long-term follow-up for managing PN will help achieve satisfactory results and minimize complications.

## Declaration

The authors declare that the work described has not been published previously and that it is not under consideration for publication elsewhere, that its publication is approved by all authors and tacitly or explicitly by the responsible authorities where the work was carried out, and that if accepted, it will not be published elsewhere including electronically in the same form, in English or any other language, without the written consent of the copyright holder.
